# An Investigation on the Feasibility of Uncalibrated and Unconstrained Gaze Tracking for Human Assistive Applications by Using Head Pose Estimation

**DOI:** 10.3390/s140508363

**Published:** 2014-05-12

**Authors:** Dario Cazzato, Marco Leo, Cosimo Distante

**Affiliations:** 1 Faculty of Engineering, University of Salento, Lecce 73100, Italy; 2 National Research Council of Italy—Institute of Optics, Arnesano (LE) 73010, Italy; E-Mails: marco.leo@cnr.it (M.L.); cosimo.distante@cnr.it (C.D.)

**Keywords:** gaze estimation, head pose estimation, gaze tracking, human-computer interaction

## Abstract

This paper investigates the possibility of accurately detecting and tracking human gaze by using an unconstrained and noninvasive approach based on the head pose information extracted by an RGB-D device. The main advantages of the proposed solution are that it can operate in a totally unconstrained environment, it does not require any initial calibration and it can work in real-time. These features make it suitable for being used to assist human in everyday life (e.g., remote device control) or in specific actions (e.g., rehabilitation), and in general in all those applications where it is not possible to ask for user cooperation (e.g., when users with neurological impairments are involved). To evaluate gaze estimation accuracy, the proposed approach has been largely tested and results are then compared with the leading methods in the state of the art, which, in general, make use of strong constraints on the people movements, invasive/additional hardware and supervised pattern recognition modules. Experimental tests demonstrated that, in most cases, the errors in gaze estimation are comparable to the state of the art methods, although it works without additional constraints, calibration and supervised learning.

## 1. Introduction

Gaze tracking plays a fundamental role in understanding human attention, feelings and desires [1]. Automatic gaze tracking provides several application in the fields of human-computer interaction (HCI) and human behavior analysis, therefore several techniques and methods have been investigated in recent years. When a person is in the field of view of a static camera, gaze can give information about the focus of attention of the subject, allowing for gaze-controlled interfaces for disabled people [2], driver attention monitoring [3], pilot training [4], provision of virtual eye contact in conferences [5] or marketing strategies analysis [6].

A survey of existing works and a detailed classification of extant methods can be viewed in [7]. Most gaze tracking methods are based on Pupil Center Corneal Reflection (PCCR) technique, [8–14]. They obtain the pose of the eye using the center of pupil contour and corneal reflections (glint) on the corneal surface from point light sources, usually one or multiple infrared (IR) lights. Normally this kind of approaches are not quite appropriate for generic interactive applications since a high-resolution camera is needed and a careful calibration is required for coupling IR lights and camera. Less invasive solutions that do not use IR are also available. The work of [15] proposes a method to achieve gaze estimation from multimodal Kinect data that is invariant to head pose, but it needs a learned person-specific 3D mesh model. In [16], after a one-time personal calibration, facial features are tracked and then used to estimate the 3D visual axis, proposing a 3D geometrical model of the eye. The method requires to accurately detect eye corners in order to create a complete 3D eye model. In [17] gaze tracking is performed using a stereo approach to detect the position and the orientation of the pupil in 3D space. A calibration procedure must be provided and, moreover, the reconstruction of the elliptic eye model cannot be well defined for all the gaze orientations. A low-cost system for 2D eye gaze estimation with low-resolution webcam images is presented in [18]: binary deformable eyeball template is modeled and 2D gaze estimation is performed depending on the displacement in eye movements and after a rigid calibration procedure. A method that, after an initial calibration, enables tracking motion of user's eye and gaze by using a single webcam—in a simplified, special case, *i.e.*, when the face is still while the eyes move—is instead proposed in [19]. Valenti *et al.* [20] combine head pose and eye location informations to accurately estimate gaze track. Their results are suitable for several applications but, unfortunately, a calibration phase that makes use of a target plane is needed in order to get reference positions to extract eye gaze directionality.

All the aforesaid methods operate in constrained condition (e.g., a very short range of head pose variations) and they need a learning phase by which manually labeled data are used to train one or more classifiers. Furthermore, a calibration phase is often needed to set up the parameters performing the mapping between the real word and the computational models embedded in the algorithmic procedures. The predominant idea behind the works in this research field was that head pose estimation information can supply only a rough estimation of the human gaze. In other words, only the area of interest of the person can be retrieved and, therefore it is inadequate, if considered alone, to obtain accurate estimation of the gaze direction and to allow applications such as remote device control or in rehabilitation scenarios. For example, in [21] authors assert that the head pose contributes to about 70% of the visual gaze, and focus of attention estimation based on head orientation alone can get an average accuracy of 88.7% in a meeting application scenario. The influence that head orientation exerts on the perception of the eye-gaze direction is investigated in [22], where the authors conclude that an image-based mechanism is responsible for the influence of head profile on gaze perception, whereas the analysis of nose angle involves the processing of face features. In [23,24] head (and eventually body) pose information is used for estimating where a person is looking at. In [25,26] the visual focus of attention is recognized by evaluating only head pose information. More recently, the authors in [27] introduced a scene-specific gaze estimator for visual surveillance: it models the interactions between head motion, walking direction and appearance in order to recover gaze directions. Anyway, in the last years this point of view is being changing due to the new perspectives emerged from the exploitation of the most advanced sensorial technologies that allow the pose estimation to become more and more accurate. For example, in [28] the pioneering attempt to use head pose information extracted by a complex supervised algorithm working on 2D images in order to control a mouse is performed: the experimental results qualitatively showed the promise of the algorithm. To overcome the drawbacks of the related works, an early study on the estimation of visual focus of attention using fuzzy fusion of head rotations and eye gaze directions has been recently introduced in [29]. Two novel techniques to estimate head rotations, based on local and appearance information, are introduced and then fused in a common framework. Anyway, this framework does not focus on inferring exact gaze estimation but, rather, it detects degrees of confidence, through fuzzy logic, regarding hypotheses that a person is looking towards a specific point.

Unfortunately, no studies have still been performed on the feasibility of an accurate gaze estimator based only on head pose information. To fill this gap, in the proposed work, an innovative approach to achieve the exact position of gaze tracking ray from data acquired from a low cost depth sensor device is introduced. The proposed solution estimates the head pose of a subject freely moving on the environment, requiring only the presence of his head in the field of view of the sensor, in order to directly derive his 3D gaze ray. Neither training nor calibration phase are required to accomplish the gaze estimation task. This is another important contribution of this paper with respect to leading approaches in the state of the art. In our work, quantitative evaluations of the gaze estimation accuracy have been achieved by a large experimental phase, with several different distance ranges and different people with diverse levels of knowledge of the performing task. The remaining sections of the paper are organized as follows: Section 2 discusses the methodological steps of the proposed approach whereas the experimental setup is introduced in Section 3. Finally, the experimental outcomes are reported in Section 4, while their discussion is reported in Section 5.

## 2. Overview of the Proposed System

**Figure 1.**
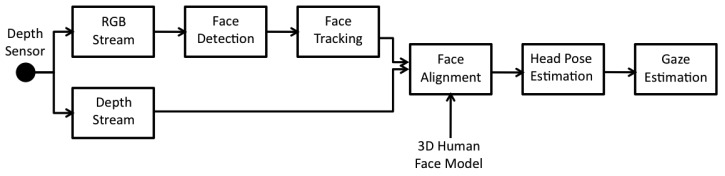
A block diagram of the proposed method.

The proposed solution works as follows: the input data are acquired from a commercial depth sensor providing as output both RGB and depth data, which are the input for the following algorithmic steps. First of all, face detection is performed on the RGB image by matching appearance with predetermined models. The detected faces are then tracked over time using local features and topological information. The available depth information is then used to iteratively match the tracked points with a 3D point cloud representing the face geometry. The 3D head pose is finally estimated in terms of yaw, pitch and roll angles, and the gaze vector is computed as the vector having the origin in the average point between the two detected 3D positions of the eyes and direction according to the estimated head pose. A block diagram of the overall system is showed in Figure 1.

### 2.1. Face Tracking and 3D Head Pose Estimation

The first algorithmic step performed on the acquired RGB images is aimed at detecting the human faces. This is done by an approach that consists of three steps [30]. A linear pre-filter is at firstly used to increase the detection speed. Then, a boosting chain [31] is applied to remove most of the non-faces from the candidate list and, finally, a color filter and a SVM filter are used to further reduce false alarms. When the system detects a face, characteristic points are identified and a parameterized face mask is automatically overlapped on the human face. After the first detection, it is then possible to track the detected face over time, reducing this way the computational load needed to process the input images. Detection of the characteristic points of the face and their temporal tracking are based on the Active Appearance Model (AAM) [32]. AAM contains a statistical model of the shape and its representation as grey-level appearance. The core of the algorithm is the matching procedure that involves finding the model parameters which minimize the difference between the given appearance and the synthesized model example, projected into the image. In order to improve tracking performances, temporal matching constraint and color information are included in the model, as suggested in [33]. The next step consists in building a 3D model of the detected face. This is done by the Iterative Closest Point (ICP) [34] technique by which a 3D point cloud model is iteratively aligned with the available 2D facial features (target). The algorithm revises the transformation, *i.e.*, combination of translation and rotation, needed to minimize the distance between the model and the target. The used 3D face model is the Candide-3 [35], a parameterized mask specifically developed for model-based coding of human faces. It allows fast reconstruction with small computing overhead. It is invariant to operating conditions and it does not depend on a specific person. This model is based on 121 linked feature points which are stored in a vector $\overset{-}{\vec{g}}$ containing their (*x, y, z*) coordinates. The model is reshaped by the equation:

(1) $${\vec{g}}_{t+1}(\sigma ,\alpha )={\vec{g}}_{t}+S\sigma +A\sigma$$

where ${\vec{g}}_{t+1}$ is the updated vector, *S* and *A* are the Shape and Animation Units and *σ, α* contain shape and animation parameters.

When the distance between the Candide-3 model and the target face is minimized the depth information of the 121 feature points is extracted from the available depth map and it will represent the input of the head pose estimation block.

The head pose estimation supplies the information about rotation angles in terms of yaw, pitch and roll, and translations, in meters, that in this paper are assumed to be expressed considering as reference point the center of the sensor. Head pose estimation is a problem with 6 Degrees of Freedom (DoF), and it can be represented with the parameter vector $\vec{p}=[\omega_{x},\omega_{y},\omega_{x},t_{x},t_{y},t_{z}]$, where *ω_x_, ω_y_, ω_z_* are the rotation parameters and *t_x_, t_y_, t_z_* are the translation parameters. They define the 3-DoF rotation matrixes *R*_3×3_ as:

(2) $$R=\left[\begin{array}{ccc} 1 & -\omega_{z} & \omega_{y}\\ \omega_{z} & 1 & -\omega_{x}\\ -\omega_{y} & \omega_{x} & 1 \end{array}\right]$$

and the 3-DoF translation vector *T*_3×1_ as:

(3) $$T=\left[\begin{array}{c} t_{x}\\ t_{y}\\ t_{z} \end{array}\right]$$

The rigid motion of a head point $\vec{X}={\left[x,y,z\right]}^{\text{T}}$ between time *t* and time *t* + 1 is: $\vec{X}(t+1)=M\times \vec{X}(t)$, where M is defined in [36] as:

(4) $$M=\left[\begin{array}{cc} R & T\\ 0 & 1 \end{array}\right]$$

Let point $\vec{X}(t)$ be projected on the image plane in $\vec{u}={\left[u_{x}u_{y}\right]}^{\text{T}}$. The explicit representation of the perspective projection function in terms of the rigid motion vector parameters and the coordinates of the point at *t* + 1 is:

(5) $$\vec{u}(t+1)=\left[\begin{array}{c} x-y\omega_{z}+z\omega_{y}+t_{x}\\ x\omega_{z}+y-z\omega_{x}+t_{y} \end{array}\right]\cdot \frac{f_{L}}{-x\omega_{y}++y\omega_{x}+z+t_{z}}(t)$$

where *f_L_* is the focal length.

In order to fuse rotation and translation information into the Candide-3 model, Equation (1) is modified as:

(6) $$\vec{g}(\sigma ,\alpha )=Rs(\overset{-}{\vec{g}}+S\sigma +A\alpha )+T$$

where *s* represents the scale. Thus, vector $\vec{p}$ has now components:

(7) $$\vec{p}=[\omega_{x},\omega_{y},\omega_{z},s,t_{x},t_{y},t_{z},\sigma ,\alpha ]$$

At the end, the position of the user's head is expressed in world coordinate X, Y, and Z which are reported based on a right-handed coordinate system with the origin at the sensor, Z pointed towards the user and Y pointed up.

**Figure 2.**
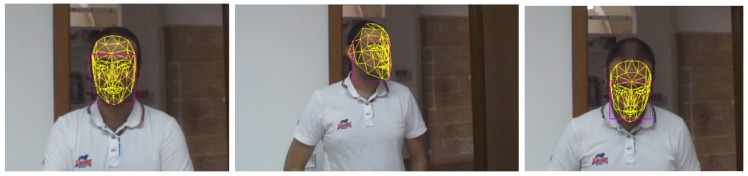
Three different snapshots of the face tracking module.

Figure 2 shows the 3D mask overlapped to the 2D facial image in three different frames. From the figure it is possible to observe that the face tracker works also in presence of non frontal views.

### 2.2. Gaze Estimation

**Figure 3.**
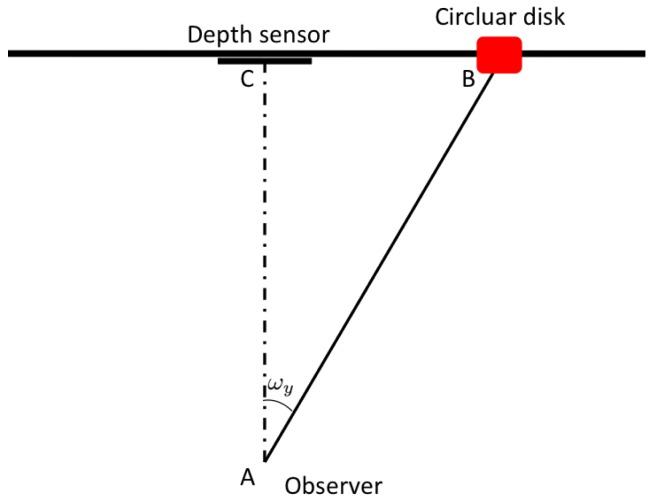
A scheme of the gaze estimation solution.

Gaze estimation step is based on the 3D model coming from the previous blocks. It geometrically models the gaze ray direction with regard to the 3D position of the sensor and thus it can be categorized as model based method. First of all, the 3D positions of the eye centers are extracted from the actual position of the overlapped 3D face model. After that, in order to define a point on the face from which the computed gaze vector takes its origin, a conventional point in the middle of the segment connecting the 3D eye center positions is taken. This point approximatively corresponds to the nose septum and it is used as the origin of the gaze track. Note that small occlusions are handled by the system, and that the eye center point is always estimated when the overlapping with the face successes. Moreover, in this way it is not necessary to use a precise pupil detector, since the computed point on the face is enough to completely solve the geometric problem. At this point, exploiting available head pose information, the direction of the gaze track is derived from the angles *ω_x_* and *ω_y_*, corresponding respectively to pitch and yaw of the vector $\vec{p}$. Then the intersection of the gaze track with a vertical plane, parallel to the image plane of the sensor, is computed. Actually, the intersection point is computed separately for the *x* and *y* axes. In Figure 3 the procedure along the *x* axis is shown and it is described in the followings. The depth sensor is able to give the information about the length of the segment $\bar{AB}$ as the component *t_z_* of the translation vector *T*. It follows that, knowing a side and an angle, we can completely solve the right-angled triangle $\hat{\text{ABC}}$ In particular $\bar{AB}=\frac{\bar{AC}}{\cos\omega_{y}}$ and $\bar{BC}=\sqrt{{\bar{AB}}^{2}-{\bar{AC}}^{2}}$. Using the same coordinate system, it is possible to compute also the cartesian equation of the gaze ray as the straight line passing for points *A* = (*x_a_, y_a_, z_a_*) and *B* = (*x_B_, y_B_, z_b_*) expressed as:

(8) $$r:\{\begin{array}{c} \frac{x-x_{A}}{x_{B}-x_{A}}=\frac{y-y_{A}}{y_{B}-y_{A}}\\ \frac{y-y_{A}}{y_{B}-y_{A}}=\frac{z-z_{A}}{z_{B}-z_{A}} \end{array}$$

with *z_a_* = 0 for the particular plane under consideration.

In case of translations on the *x* and *y* axes, the vector can be algebraical summed up with the computed value, in order to translate the gaze vector to the right position. Finally, in order to represent on a monitor the actual intersection point between the gaze vector and the plane, the world coordinates are normalized to image plane according to:

(9) $$\begin{array}{cc} x=X-\frac{L}{R-L}\cdot I_{x} & y=Y-\frac{T}{T-B} \end{array}\cdot I_{y}$$

where: (*X, Y*) and (*x, y*) are the world and image plane coordinates, respectively; *L, R, T, B* are the left, right, top and bottom bounds of the considered user's field of view; *I_x_* and *I_y_* are the width and height of the displaying area on the monitor (in pixels).

## 3. Experimental Setup

**Figure 4.**
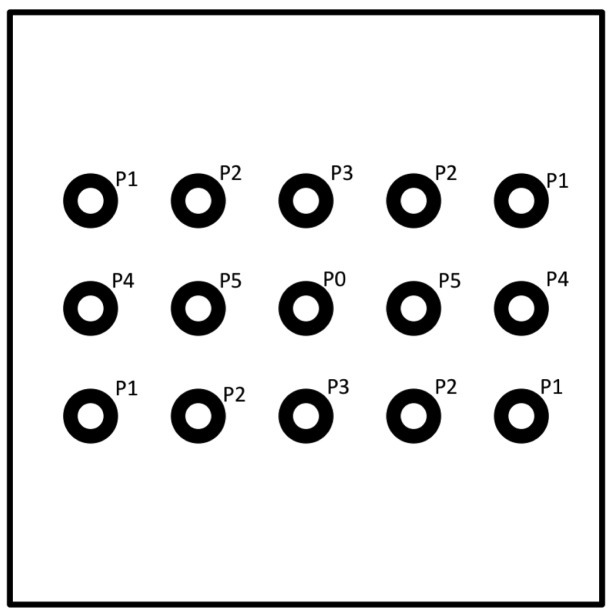
The used grouping scheme for target points during tests.

The experimental setup was defined as follows: a Microsoft Kinect device was used as depth sensor and it was positioned at a height of 150 cm from the ground. Behind the sensor a square panel (2 m per side) was positioned and 15 circular markers were stuck on it. The markers were distributed on three rows, 5 markers on each row, with a distance of 50 cm from each other. Markers were divided into subsets as showed in Figure 4 in order to group together points that presented the same distance from the sensor in terms of *x,y* or both axes, from P1 to P5, while P0 corresponds to the depth sensor position. For example, P4 are the points with a distance of 1 meter from the sensor along the x axes and aligned along y axes, and so on. The depth sensor was placed in correspondence of the marker at the centre of the panel.

**Figure 5.**
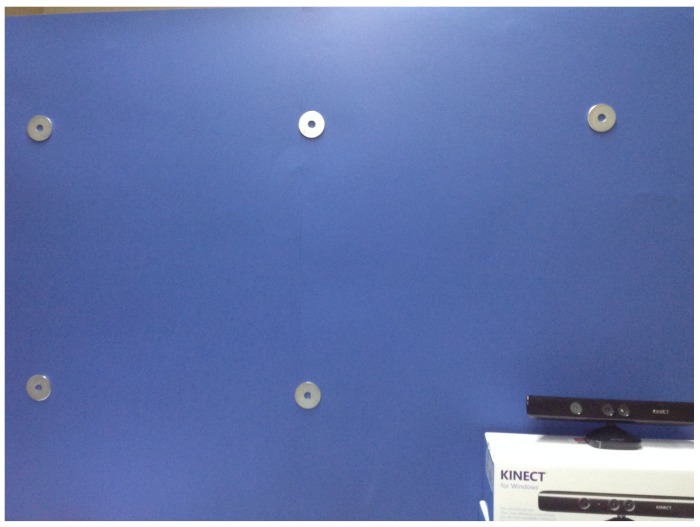
A portion of the panel used in the experimental phase.

Figure 5 shows one quarter of the panel, exactly the upper-leftmost. The 3D positions of the markers represented the ground truth information for the following experimental phase (Section 4) is devoted to the evaluation of the system's capability to estimate the points of regard of the users.

**Figure 6.**
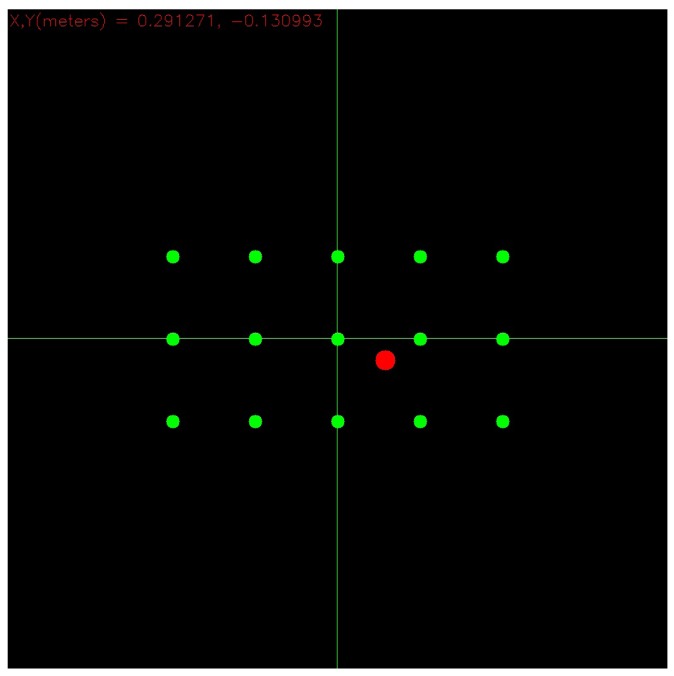
A picture of the monitor where gaze hits can be drawn.

Figure 6 shows instead a picture of the monitor where gaze hits can be drawn, making use of the Equation (9): the red circle represents the point of the panel where the user is meanwhile looking at. Green circles represent instead the projection onto the monitor of the markers placed on the panel surface. The sensor's working ranges are 43° vertical, 57° horizontal and [40 *cm*, 300 *cm*] in depth [37].

Three different distances between the user and the panel were considered in the experiments (*i.e.*, 70 cm, 150 cm and 250 cm) (The exact distance between the user and the panel was retrieved by adding 4 cm to the computed depth map in order to take into account the displacement of the physical position of the sensor with respect the plane of the panel.): in this operating conditions the head pose angles range in the intervals [−56.0 °, +56.0 ° ] for the yaw, [−35.5 °, +35.5 ° ] for the pitch and [−90.0 °, +90.0 ° ] for the roll.

**Figure 7.**
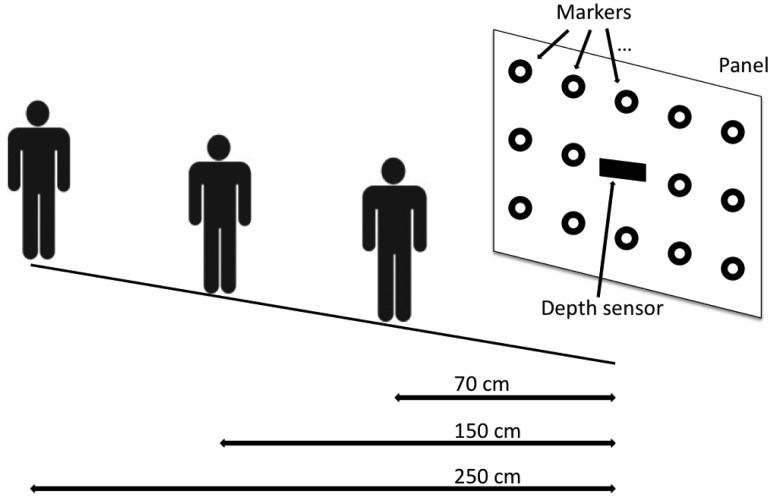
The different users positions for the experimental phase.

A scheme of the experimental setup illustrating the three different users positions is shown in Figure 7. Notice that, the above operating ranges allow the user to freely move the head in all the directions and in particular, despite of most of the state-of-the-art methods making use of the Viola-Jones face detector [31], also large rolling movements can be handled.

## 4. Experimental Results

The proposed method was tested with nine different persons. According to different similar works in this research field (for example in [38]), in order to get a comprehensive study, persons were divided into three groups, three persons for each group. The first group was composed by experienced persons, *i.e.*, persons that knew how the system works and that already had tried the system before the test session. The second group was composed by persons that were trying the system for the first time but that had been informed about how the system works. In light of that knowledge, it was more probable that they would have moved the head even in case of sequential pointing of close markers onto the panel. Finally, in the last group there were unaware people who were just placed in front of the sensor and they were asked to point towards the markers. No constraints were given to the participants in terms of eyeglasses, beard or hairstyle and, in order to allow for wild settings, no panel or uniform background color were put behind the participants. These three experimental benchmarks permitted to verify the system's accuracy in relation to different levels of awareness of the users, which may be encountered in different applicative contexts.

The experiment was made as follows for all three groups: persons were asked to look at each of the markers onto the panel, in a predefined order. The gaze direction relative to a given marker, was that one estimated by the system when the person confirmed, by an oral feedback, that the marker was its point of regard. The estimated gaze direction was projected onto the panel and then compared with manually computed ground truth data. Therefore the errors were measured as the distances between the estimated intersection points and the ground truth data. The errors were expressed both in centimeters, as well as by the difference between the angles described by the estimated and ground truth rays.

The outcomes of the experimental tests are shown in Tables 1–3. The first column reports the labels of the markers under consideration (see Section 3 for label assignment) whereas the second column shows the tested distances, *i.e.*, 70 cm, 150 cm and 250 cm. Errors were computed separately for each group and for yaw and pitch angles. Also error standard deviation, reported in degrees, was taken into account and reported in tables. Note that “n.a.” stands for not available data, corresponding to missing overlapping of the Candide-3 model on the current face.

From Table 1, it can be observed that the results for the first group of persons were very accurate: the average error was at maximum about 3 degrees, encountered when the persons stand at 70 cm from the panel, with a standard deviation of the error that was about 1.5 degrees in both directions. This is the prove that the proposed system is well suited for those application contexts where the users can be learned to exploit at best its functionalities: e.g., remote control of the device's cursor in cases of physical impairments. Table 2 demonstrates that the system reported encouraging results also on the second benchmark of persons, *i.e.*, the informed ones: the average errors slightly increased, compared with those achieved for the group 1, to about 4.5 degrees and some peaks in the standard deviation were accounted due to the slowness of the corresponding person to become familiar with the system. The results reported on this group demonstrated that the proposed system can be exploited in all those application contexts where users can be preliminarily informed about the right modalities to interact (e.g., for gaming purposes, where a quick information session generally precedes the start of the game).

Finally, from Table 3, reporting the results on the third group of persons , it is possible to derive that the errors in accuracy still remained under 12 degrees (often around 5 degrees) with a standard deviation ranging from 2 to 6 degrees. In our opinion, this is a very interesting result considering that this group of persons are completely unaware about the system. This demonstrates that the proposed system can be exploited also in those applications where it is not possible to constraint the user behavior, e.g., in assistive applications involving persons with neurological impairments or audience measurements.

Notice also that the gaze estimation accuracy, independently from the benchmark under investigation, remained satisfactory even when the user's distance increased from the depth sensor (in particular the errors encountered at a distance of 150 cm were very encouraging). By our knowledge, this paper represents the first investigation of the performance of a gaze estimation in those challenging conditions: all the state-of-the-art methods, in fact, reports the accuracy achieved at a distance less than 1 m between user and target. In light of this, in order to evaluate the accuracy of the proposed method with respect the leading approaches in the literature, a further experiment was performed. In this further experiment the testing scenario mostly used in the promising works in the literature was set up. In that scenario the users are put in front of a screen, at a distance in the range [54 *cm*, 67 *cm*], and they are asked to observe a list of point on the screen.

In Table 4 the errors in estimating the gaze direction, of the proposed approach and of the leading methods mentioned in Section 1, are reported. Here we point out again that all the compared approaches make use of supervised algorithms and/or invasive device and/or a calibration and/or they limit the head movements.

From the table, it can be seen that in the case of experienced or informed users, only the state-of-the-art methods making use of additional training and/or calibration phases outperform in accuracy the proposed approach. In the case of unaware users, the experienced accuracy is a little bit lower than that of some comparing approaches but, it remains well suited for most of the main attractive application domains for gaze tracking systems (e.g., remote rehabilitation, healthcare, monitoring, audience measurement, *etc.*). This is a very encouraging results that open a new way to deal with the gaze estimation issue: it demonstrated that new sensorial technologies combined with robust head pose estimation algorithms can bring to a relaxation of the environmental constraints and to a simplification of the algorithmic steps involved in the gaze estimation approaches.

During the above experimental phases a further evaluation was done on the users in groups 1 and 2 in order to check the actual possibility of using the proposed system to remotely control a device. After each experimental session, each person was asked to look at a screen and to try to control the mouse pointer by using his gaze and finally to give a feedback about usability and familiarity. All of the participant feel comfortable and able to easily use the system as a control device.

Concerning computational remarks, the involved algorithms have been implemented using Microsoft Visual C++ developing environment and, running on an Ultrabook Intel i3 CPU @ 1.8 GHz with 4 GB of RAM, with RGB and depth images taken at a resolution of 640 × 480, 30 fps, during the experimental phase the system was able to work in real-time.

## 5. Conclusions

This work presented an investigation on the feasibility of a gaze estimation system working in an unconstrained and noninvasive environment and that does not require any additional hardware (like IR light sources, wearable devices, *etc.*). The proposed solution makes use of a low cost commercial depth sensor and it estimates head pose information by combining RGB and depth data. This method has then been tested with both trained and untrained persons in an unconstrained setting, and errors have been quantitatively measured. In addition, it has been also carefully compared with the leading approaches in the state of the art, showing that their errors are comparable even if the considered approaches work well only under particular conditions and/or if specialized hardware (sometime invasive) is available. For this reason, the proposed approach is more suitable for most of the main attractive application domains concerning unconstrained gaze tracking systems like remote rehabilitation, therapy supply or ambient assistive living. Another advantage of the proposed solution is that it makes use of commercial hardware and no calibration phase is required. This could make it exploitable also from non experts user and then it can become a technological support in related research fields, for example for studying social human behaviors or being used in socially assistive robotics during human-robot interaction. Furthermore, we are aware that in particular application domains, e.g., for studying particular neurological diseases or to realize hand-free control of mobile devices, is also indispensable to combine head pose information with an accurate pupil center locator and this investigation will be the subject of our future research works.

## Figures and Tables

**Table 1.** Experiments with the first group of experienced persons: they knew how the system works and that already tried the system before the test session.

		**Errors**					
		**x (cm)**	**x (deg)**	**y (cm)**	**y (deg)**	**Std x (deg)**	**Std y (deg)**
P0	70 cm	1.50	1.22	2.66	2.18	0.34	0.87
	150 cm	3.50	1.33	4.83	1.84	0.54	0.93
	250 cm	6.00	1.37	8.50	1.94	0.67	0.85
P1	70 cm	n.a.	n.a.	n.a.	n.a.	n.a	n.a
	150 cm	6.00	1.61	8.00	2.79	0.93	1.36
	250 cm	2.60	0.52	4.00	0.88	0.20	0.37
P2	70 cm	8.77	5.03	7.66	4.37	2.36	2.09
	150 cm	0.16	0.05	11.83	4.15	0.01	1.66
	250 cm	4.33	0.95	5.83	1.29	0.38	0.52
P3	70 cm	5.61	4.58	3.50	1.94	2.33	1.06
	150 cm	6.83	2.60	8.83	3.08	0.98	1.20
	250 cm	4.66	1.06	1.83	0.40	0.44	1.50
P4	70 cm	n.a.	n.a.	n.a.	n.a.	n.a.	n.a.
	150 cm	0.50	0.13	9.33	3.56	0.07	1.95
	250 cm	0.66	0.13	15.66	3.47	0.49	1.97
P5	70 cm	3.66	2.03	3.83	3.13	1.11	1.58
	150 cm	0.33	0.11	4.16	1.59	0.04	0.60
	250 cm	4.33	0.95	8.16	1.87	0.36	0.97
Total Averages	70 cm	4.88	3.22	4.41	2.90	1.53	1.40
	150 cm	2.88	0.97	7.83	2.83	0.42	1.28
	250 cm	3.77	0.83	7.25	1.64	0.42	1.03

**Table 2.** Experiments with the second group persons that were trying the system for the first time but that have been informed how it works.

		**Errors**					
		**x (cm)**	**x (deg)**	**y (cm)**	**y (deg)**	**Std x (deg)**	**Std y (deg)**
P0	70 cm	2.50	2.04	2.33	1.90	0.53	0.57
	150 cm	6.24	2.38	7.41	2.83	0.95	1.09
	250 cm	28.5	6.50	13	2.97	1.95	1.18
P1	70 cm	n.a.	n.a.	n.a.	n.a.	n.a.	n.a.
	150 cm	2.00	0.53	21.58	7.70	0.30	3.61
	250 cm	19.00	3.84	27.00	6.05	2.30	2.96
P2	70 cm	10.16	5.89	17.16	10.40	2.59	5.30
	150 cm	5.83	1.89	19.08	6.78	0.92	3.45
	250 cm	3.00	1.50	0.65	0.33	0.79	0.33
P3	70 cm	2.83	2.31	8.33	4.77	1.01	2.85
	150 cm	15.83	6.02	13.33	4.69	3.99	2.83
	250 cm	18.5	4.23	20.5	4.58	1.98	2.12
P4	70 cm	n.a.	n.a.	n.a.	n.a.	n.a.	n.a.
	150 cm	4.75	1.27	15.08	5.74	0.77	2.91
	250 cm	33.00	6.79	12.50	2.86	3.66	2.01
P5	70 cm	11.16	0.83	6.51	0.68	0.36	0.29
	150 cm	7.50	2.53	3.00	1.14	1.10	0.59
	250 cm	22.50	5.03	19.00	4.34	2.73	1.52
Total Averages	70 cm	6.66	4.19	7.16	4.44	1.12	2.25
	150 cm	6.98	2.44	13.25	4.81	1.33	2.41
	250 cm	20.75	4.51	15.58	3.52	2.23	1.68

**Table 3.** Experiments with the third group of persons that were totally unaware of how the system works.

		**Errors**					
		**x (cm)**	**x (deg)**	**y (cm)**	**y (deg)**	**Std x (deg)**	**Std y (deg)**
P0	70 cm	4.00	3.27	0.00	0.00	1.92	0.00
	150 cm	15.00	5.71	10.00	3.81	3.01	2.19
	250 cm	71.00	12.00	15.85	2.74	8.86	2.03
P1	70 cm	n.a.	n.a.	n.a.	n.a.	n.a.	n.a.
	150 cm	17.00	4.73	29.00	10.46	3.15	7.61
	250 cm	62.00	13.15	34.00	7.64	7.96	4.19
P2	70 cm	24.99	23.13	34.61	23.13	12.17	14.01
	150 cm	1.78	0.61	10.90	3.82	0.49	1.38
	250 cm	27.00	6.05	17.00	3.79	3.42	1.83
P3	70 cm	24.61	19.37	10.10	5.11	10.33	2.91
	150 cm	33.20	12.48	17.10	6.06	7.08	3.20
	250 cm	90.23	19.84	12.6	2.80	10.05	1.18
P4	70 cm	n.a.	n.a.	n.a.	n.a.	n.a.	n.a.
	150 cm	3.90	1.01	4.80	1.83	0.73	0.81
	250 cm	12.54	2.52	24.67	5.63	1.89	3.21
P5	70 cm	19.80	4.10	12.20	3.35	1.87	1.79
	150 cm	2.78	0.96	17.38	6.60	0.49	4.03
	250 cm	4.44	0.98	24.24	5.53	0.41	3.31
Total Averages	70 cm	18.35	12.67	12.20	7.90	6.57	4.67
	150 cm	12.27	4.25	14.86	5.43	2.49	3.20
	250 cm	44.61	9.75	20.75	4.69	5.43	2.62

**Table 4.** Head motion ranges (in world coordinate system) used in the final evaluation.

**Method**	**Category**	**Reported Error**	**Camera(s)**	**Additional Requirements**
Proposed (experienced users)	Model	3.1°	Depth Sensor	None
Proposed (informed users)	Model	3.6°	Depth Sensor	None
Proposed (unaware users)	Model	6.9°	Depth Sensor	None
Lu *et al.* [39]	Appearance	2–3°	1	Capture video
Sugano *et al.* [40]	Appearance	4–5°	1	≈ 10^3^ training samples
Nakazawa and Nitschke [10]	Model	0.9°	1 IR	IR LEDs & projector
Villanueva and Cabeza [11]	Model	1°	1 IR	2–4 IR LEDs
Zhu and Ji [12]	Model	2°	2 IR	n IR LEDs
Guestrin and Eizenman [41]	Model	1–3°	1 IR	2 IR LEDs
Yoo and Chung [13]	Model	1–2.5°	2 IR	5 IR LEDs
Noureddin *et al.* [14]	Model	1–3°	2–4 IR	IR LEDs + Mirrors
